# Supplementary material for: The structural basis of odorant recognition in insect olfactory receptors
Source: Nature. 2021 Aug 4;597(7874):126–31. doi: 10.1038/s41586-021-03794-8 (PMC8410599; doi:10.1038/s41586-021-03794-8)
Supplement: Supplementary file 1 — This file contains supplementary text, supplementary figure 1 and supplementary tables 1 – 11. [file 41586_2021_3794_MOESM1_ESM.pdf]

nature portfolio
https://doi.org/10.1038/s41586-021-03794-8

Supplementary information

The structural basis of odorant recognition in insect olfactory receptors

In the format provided by the authors and unedited

Supplementary Information Table of Contents

Page Number	Supplementary Information	Description
2	Figure 1	Uncropped Western blots.
3-4	Table 1	MhOR5 receptor response to a panel of ligands.
5	Table 2	Wild-type and mutant MhOR5 receptor response to eugenol.
6	Table 3	Wild-type and mutant Orco/OR heteromer responses.
6	Table 4	Wild-type and mutant MhOR5 receptor response to DEET.
7-9	Table 5	MhOR5 M209V and I213M receptor response to a panel of odorants.
10-11	Table 6	MhOR1 receptor response to a panel of ligands.
12-13	Table 7	Lifetime sparseness values for olfactory receptors.
14-15	Table 8	Ordering of compounds in MhOR5 tuning curves.
16-17	Table 9	Molecular descriptors of ligands used in multiple regression analysis.
18	Table 10	Wild-type MhOR1 and putative binding-pocket mutant responses.
19	Table 11	Wild-type MhOR1 and M231I receptor response to a panel of odorants.

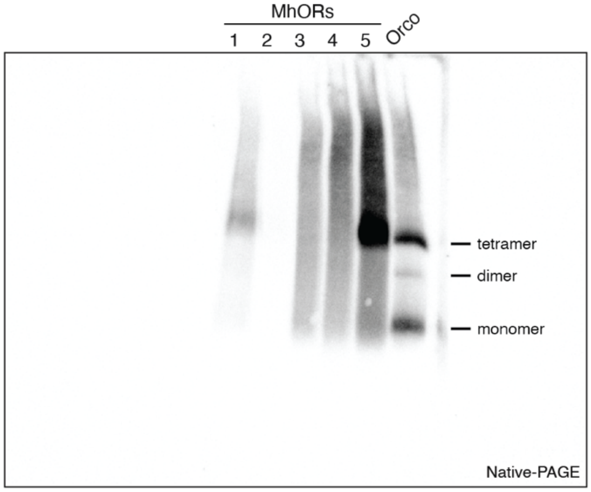

as seen in Extended Data Figure 1a

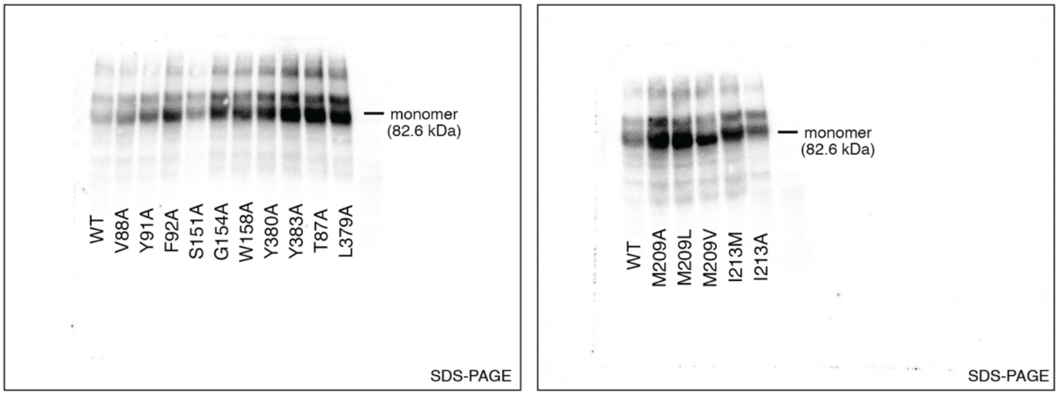

as seen in Extended Data Figure 9g (left)

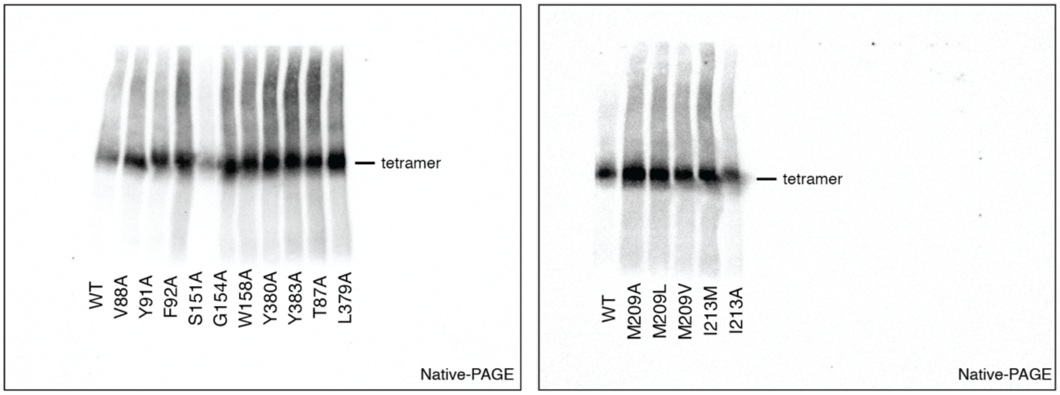

as seen in Extended Data Figure 9g (right)

Supplementary Figure 1. Uncropped Western blots with anti-GFP staining. Positions of the tetramer, dimer, and monomer are shown as applicable. In the Native-PAGE gel in Extended Data Figure 1a, Orco serves as the control condition. In all gels shown in Extended Data Figure 9, the wild-type MhOR5 serves as the control, to which mutant channel expression can be compared.

Ligand	CAS #	Activity Index	log(EC_{50})	max ΔF/F	N
1-hexanol	111-27-3	4.202 ± 0.156	-4.324 ± 0.047	0.972 ± 0.036	10
1-octanol	111-87-5	4.661 ± 0.140	-4.985 ± 0.033	0.935 ± 0.026	13
(*R*)-1-octen-3-ol	3391-86-4	4.692 ± 0.324	-4.931 ± 0.131	0.953 ± 0.061	12
1-pentanol	71-41-0	1.434 ± 0.062	-2.000 ± 0.000	0.717 ± 0.031	11
2-acetylthiophene	88-15-3	4.545 ± 0.178	-5.245 ± 0.079	0.868 ± 0.034	14
2-ethylphenol	90-00-6	5.143 ± 0.211	-5.062 ± 0.109	1.015 ± 0.033	15
2-heptanone	110-43-0	4.733 ± 0.233	-4.956 ± 0.080	0.955 ± 0.045	11
2-undecanone	112-12-9	0.437 ± 0.082	-2.000 ± 0.000	0.218 ± 0.041	7
2,3-butanediol	513-85-9	0.357 ± 0.061	-2.000 ± 0.000	0.178 ± 0.030	3
2,4,5-trimethylthiazole	13623-11-5	4.641 ± 0.173	-4.875 ± 0.062	0.954 ± 0.038	15
3-octanol	589-98-0	4.856 ± 0.144	-5.018 ± 0.045	0.968 ± 0.029	13
4-ethylphenol	123-07-9	5.177 ± 0.202	-5.158 ± 0.070	1.003 ± 0.036	15
4-methoxyphenylacetone	122-84-9	4.867 ± 0.188	-4.887 ± 0.079	0.994 ± 0.030	13
acetaldehyde diethyl acetal	105-57-7	1.169 ± 0.133	-2.000 ± 0.000	0.584 ± 0.067	7
acetic acid	64-19-7	0.265 ± 0.081	-2.000 ± 0.000	0.133 ± 0.040	4
acetophenone	98-86-2	5.265 ± 0.162	-5.487 ± 0.052	0.961 ± 0.031	13
alpha-pinene	7785-70-8	4.188 ± 0.151	-4.314 ± 0.047	0.972 ± 0.036	11
L-(+)-arabinose	5328-37-0	0.202 ± 0.062	-2.000 ± 0.000	0.101 ± 0.031	4
benzaldehyde	100-52-7	5.314 ± 0.291	-4.976 ± 0.080	1.068 ± 0.055	13
butyl acetate	123-86-4	5.107 ± 0.273	-5.047 ± 0.068	1.014 ± 0.054	13
butyric acid	107-92-6	0.500 ± 0.088	-2.000 ± 0.000	0.250 ± 0.044	11
caffeine	58-08-2	0.108 ± 0.043	-2.000 ± 0.000	0.054 ± 0.021	4
citric acid	77-92-9	0.175 ± 0.054	-2.000 ± 0.000	0.087 ± 0.027	4
decanal	112-31-2	1.399 ± 0.063	-2.000 ± 0.000	0.700 ± 0.032	11
DEET	134-62-3	3.114 ± 0.155	-4.298 ± 0.061	0.724 ± 0.035	12
denatonium benzoate	3734-33-6	-0.035 ± 0.012	-3.963* ± 0.056	-0.009 ± 0.003	7
ethyl acetate	141-78-6	0.707 ± 0.096	-2.000 ± 0.000	0.354 ± 0.048	7
ethyl butyrate	105-54-4	4.161 ± 0.191	-4.147 ± 0.114	1.006 ± 0.041	14
ethyl hexanoate	123-66-0	5.060 ± 0.191	-5.129 ± 0.091	0.983 ± 0.027	17
eugenol	97-53-0	5.701 ± 0.026	-5.702 ± 0.018	1.000 ± 0.000	122
(±)-geosmin	16423-19-1	3.677 ± 0.162	-4.308 ± 0.071	0.855 ± 0.040	11
D-(+)-glucose	50-99-7	0.232 ± 0.101	-2.000 ± 0.000	0.116 ± 0.051	4
heptanoic acid	111-14-8	0.562 ± 0.086	-2.000 ± 0.000	0.281 ± 0.043	12
hexanal	66-25-1	4.228 ± 0.249	-4.480 ± 0.097	0.943 ± 0.051	11
indole	120-72-9	5.053 ± 0.280	-5.106 ± 0.139	0.982 ± 0.036	13
isobutyl acetate	110-19-0	4.552 ± 0.159	-4.705 ± 0.076	0.967 ± 0.030	14
isopropyl tiglate	1733-25-1	4.357 ± 0.180	-4.669 ± 0.061	0.934 ± 0.039	13
L-(+)-lactic acid	79-33-4	0.419 ± 0.094	-2.000 ± 0.000	0.210 ± 0.047	10
(*R*)-(+)-limonene	5989-27-5	4.964 ± 0.141	-5.539 ± 0.115	0.899 ± 0.025	13
linalool	78-70-6	4.111 ± 0.139	-4.572 ± 0.053	0.899 ± 0.026	14
methyl benzoate	93-58-3	5.420 ± 0.334	-5.348 ± 0.124	1.010 ± 0.052	13
methyl hexanoate	106-70-7	4.907 ± 0.252	-5.075 ± 0.112	0.967 ± 0.043	14
methyl laurate	111-82-0	0.346 ± 0.064	-2.000 ± 0.000	0.173 ± 0.032	11

L-glutamic acid monosodium salt monohydrate (MSG)	6106-04-3	0.283 ± 0.092	-2.000 ± 0.000	0.141 ± 0.046	4
n-caproic acid	142-62-1	0.641 ± 0.140	-2.000 ± 0.000	0.321 ± 0.070	7
o-cresol	95-48-7	4.277 ± 0.164	-4.550 ± 0.053	0.942 ± 0.038	13
octanoic acid	124-07-2	0.794 ± 0.097	-2.000 ± 0.000	0.397 ± 0.049	12
prenyl acetate	1191-16-8	4.197 ± 0.174	-4.542 ± 0.071	0.928 ± 0.043	12
propyl acetate	109-60-4	4.103 ± 0.207	-4.198 ± 0.046	0.977 ± 0.047	11
sucrose	57-50-1	0.215 ± 0.061	-2.000 ± 0.000	0.108 ± 0.030	4
sulcatone	110-93-0	4.682 ± 0.228	-4.773 ± 0.060	0.982 ± 0.047	13
thiazole	288-47-1	0.899 ± 0.097	-2.000 ± 0.000	0.450 ± 0.049	10
trans-3-hexen-1-ol	928-97-2	1.719 ± 0.054	-2.000 ± 0.000	0.859 ± 0.027	10
VUAA1	525582-84-7	-0.105 ± 0.053	-4.814* ± 0.141	-0.022 ± 0.012	7

Supplementary Table 1 l MhOR5 receptor response to a panel of odorants, tastants and synthetic ligands, assayed in the functional GCaMP assay (Figs. 1, 4, Extended Data Figs. 1, 2). Activity Index, log(EC_{50}) and max $\Delta F/F$ are three metrics used to characterized the dose-response curve for each ligand. The Activity Index is defined as the negative product of log(EC_{50}) and max $\Delta F/F$. EC_{50} is the concentration of ligand at which the response reaches the midpoint. max $\Delta F/F$ is the maximum response achieved. For the following supplementary tables, these measures are defined in the same manner. All values are shown with SEM.

Mutation	GCaMP Normalized Baseline	Activity Index	log(EC_{50})	max ΔF/F	N
wild-type MhOR5	2.201 ± 0.092	5.701 ± 0.026	-5.702 ± 0.018	1.000 ± 0.000	122
T87A	1.511 ± 0.195	6.635 ± 0.385	-5.590 ± 0.058	1.186 ± 0.064	7
V88A	1.196 ± 0.092	6.817 ± 0.503	-4.907 ± 0.048	1.391 ± 0.108	6
Y91A	1.275 ± 0.129	9.811 ± 0.588	-4.589 ± 0.061	2.133 ± 0.107	6
F92A	4.152 ± 0.311	0.545 ± 0.035	-2.000 ± 0.000	0.272 ± 0.018	7
S151A	5.227 ± 0.437	0.098 ± 0.010	-2.000 ± 0.000	0.049 ± 0.005	6
G154A	2.486 ± 0.249	0.583 ± 0.053	-2.000 ± 0.000	0.291 ± 0.026	6
W158A	1.208 ± 0.134	0.167 ± 0.029	-2.000 ± 0.000	0.084 ± 0.014	6
M209A	5.701 ± 0.240	0.218 ± 0.047	-2.000 ± 0.000	0.109 ± 0.024	7
M209V	1.671 ± 0.087	5.075 ± 0.292	-4.707 ± 0.046	1.078 ± 0.062	6
M209L	1.184 ± 0.155	9.586 ± 0.873	-5.000 ± 0.026	1.939 ± 0.180	6
I213A	2.418 ± 0.781	0.375 ± 0.043	-2.000 ± 0.000	0.187 ± 0.022	6
I213M	2.006 ± 0.089	6.362 ± 0.865	-5.046 ± 0.042	1.258 ± 0.169	7
Y362A	1.330 ± 0.216	8.120 ± 0.748	-4.655 ± 0.031	1.741 ± 0.154	6
Y362F	1.752 ± 0.088	5.255 ± 0.264	-5.606 ± 0.187	0.944 ± 0.061	6
L379A	1.481 ± 0.126	5.192 ± 0.305	-5.590 ± 0.071	0.932 ± 0.063	6
Y380A	8.025 ± 0.947	0.115 ± 0.005	-2.000 ± 0.000	0.057 ± 0.003	6
Y383A	1.129 ± 0.094	0.282 ± 0.035	-2.000 ± 0.000	0.141 ± 0.017	6
L465A	1.168 ± 0.116	0.441 ± 0.051	-2.000 ± 0.000	0.221 ± 0.025	6
Q467A	1.311 ± 0.142	0.083 ± 0.011	-2.000 ± 0.000	0.041 ± 0.005	7
Q467E	8.386 ± 1.014	0.108 ± 0.018	-2.000 ± 0.000	0.054 ± 0.009	6
Q467R	1.164 ± 0.093	0.856 ± 0.185	-2.000 ± 0.000	0.428 ± 0.092	7
Q467N	1.220 ± 0.103	7.810 ± 0.643	-5.269 ± 0.137	1.472 ± 0.087	6
V468A	2.924 ± 0.285	4.475 ± 0.199	-5.779 ± 0.131	0.778 ± 0.047	6
V468Q	1.943 ± 0.274	7.944 ± 0.568	-5.390 ± 0.049	1.472 ± 0.100	6

Supplementary Table 2 l Response of wild-type and mutant MhOR5 receptors to eugenol, assayed in the functional GCaMP assay (Figs. 2-4, Extended Data Figs. 9,10). Baseline fluorescence is divided by the negative GCaMP-only control baseline fluorescence on the same plate. All values are shown with SEM.

Construct	Ligand	Activity Index	log(EC_{50})	max ΔF/F	N
A. bakeri Orco	VUAA1	4.258 ± 0.030	-4.258 ± 0.030	1.000 ± 0.000	8
A. bakeri Orco Q472A	VUAA1	0.239 ± 0.068	-2.000 ± 0.000	0.120 ± 0.034	5
A. bakeri Orco / *A. gambiae* OR28	acetophenone	4.846 ± 0.081	-4.846 ± 0.081	1.000 ± 0.000	7
A. bakeri Orco Q472A / *A. gambiae* OR28	acetophenone	4.290 ± 0.652	-3.989 ± 0.053	1.068 ± 0.152	7
A. bakeri Orco	acetophenone	0.122 ± 0.039	-2.000 ± 0.000	0.061 ± 0.019	4
A. gambiae OR28	acetophenone	0.070 ± 0.009	-2.000 ± 0.000	0.035 ± 0.005	4

Supplementary Table 3 l Response of wild-type and mutant *A. bakeri* Orco and *A. gambiae* OR heteromers tested with their cognate ligands in the functional GCaMP assay (Fig. 2). Max ΔF/F is normalized to respective wild-type heteromer. All values are shown with SEM.

Mutation	GCaMP Normalized Baseline	Activity Index	log(EC_{50})	max ΔF/F	N
wild-type MhOR5	1.837 ± 0.157	3.114 ± 0.155	-4.298 ± 0.061	0.724 ± 0.035	12
M209A	3.965 ± 0.364	0.895 ± 0.122	-5.243 ± 0.100	0.173 ± 0.026	9
M209V	1.484 ± 0.210	6.654 ± 0.320	-5.122 ± 0.080	1.301 ± 0.065	6
M209L	1.027 ± 0.122	6.057 ± 1.031	-4.158 ± 0.030	1.452 ± 0.242	8
I213A	4.962 ± 1.131	0.323 ± 0.056	-2.000 ± 0.000	0.162 ± 0.028	7
I213M	1.807 ± 0.230	0.042 ± 0.007	-2.000 ± 0.000	0.021 ± 0.004	6

Supplementary Table 4 l Response of wild-type and mutant MhOR5 receptors to DEET assayed in the functional GCaMP assay (Fig. 4 and Extended Data Fig. 9). Max ΔF/F is normalized to wild-type MhOR5 with DEET. Baseline fluorescence is divided by the negative GCaMP-only control baseline fluorescence on the same plate. All values are shown with SEM.

a)

Odorant	Activity Index	log(EC_{50})	max ΔF/F	N
eugenol	5.075 ± 0.292	-4.707 ± 0.046	1.078 ± 0.062	6
methyl benzoate	3.764 ± 0.351	-4.190 ± 0.034	0.899 ± 0.086	5
benzaldehyde	4.273 ± 0.327	-3.679 ± 0.019	1.161 ± 0.088	6
acetophenone	4.178 ± 0.359	-4.246 ± 0.040	0.983 ± 0.081	6
4-ethylphenol	4.935 ± 0.439	-4.259 ± 0.109	1.158 ± 0.101	6
2-ethylphenol	5.261 ± 0.327	-4.376 ± 0.023	1.202 ± 0.072	6
butyl acetate	5.069 ± 0.439	-3.903 ± 0.054	1.295 ± 0.104	6
ethyl hexanoate	1.184 ± 0.206	-2.000 ± 0.000	0.592 ± 0.103	6
indole	6.108 ± 0.370	-4.282 ± 0.043	1.425 ± 0.080	6
(*R*)-(+)-limonene	5.945 ± 0.234	-5.101 ± 0.037	1.166 ± 0.045	6
methyl hexanoate	5.049 ± 0.265	-4.095 ± 0.053	1.231 ± 0.054	6
4-methoxyphenylacetone	6.422 ± 0.290	-4.527 ± 0.041	1.419 ± 0.064	6
3-octanol	4.333 ± 0.225	-3.890 ± 0.041	1.113 ± 0.054	6
2-heptanone	5.141 ± 0.308	-4.073 ± 0.064	1.261 ± 0.068	6
(*R*)-1-octen-3-ol	5.066 ± 0.254	-3.963 ± 0.059	1.275 ± 0.048	6
sulcatone	5.429 ± 0.242	-4.226 ± 0.072	1.287 ± 0.063	6
1-octanol	3.215 ± 0.266	-4.078 ± 0.037	0.789 ± 0.066	6
2,4,5-trimethylthiazole	1.733 ± 0.141	-2.000 ± 0.000	0.867 ± 0.070	6
isobutyl acetate	4.824 ± 0.387	-3.765 ± 0.033	1.282 ± 0.105	6
2-acetylthiophene	2.622 ± 0.276	-3.777 ± 0.048	0.696 ± 0.075	5
isopropyl tiglate	4.174 ± 0.235	-3.983 ± 0.044	1.050 ± 0.067	5
o-cresol	2.036 ± 0.116	-2.000 ± 0.000	1.018 ± 0.058	6
hexanal	1.536 ± 0.187	-2.000 ± 0.000	0.768 ± 0.094	5
1-hexanol	1.325 ± 0.145	-2.000 ± 0.000	0.662 ± 0.073	5
prenyl acetate	4.806 ± 0.248	-3.990 ± 0.024	1.205 ± 0.065	6
alpha-pinene	3.272 ± 0.454	-3.779 ± 0.066	0.864 ± 0.115	5
ethyl butyrate	1.410 ± 0.169	-2.000 ± 0.000	0.705 ± 0.084	6
linalool	3.670 ± 0.106	-3.991 ± 0.042	0.920 ± 0.028	6
propyl acetate	0.864 ± 0.196	-2.000 ± 0.000	0.432 ± 0.098	5
(±)-geosmin	0.199 ± 0.034	-2.000 ± 0.000	0.099 ± 0.017	6
DEET	6.654 ± 0.320	-5.122 ± 0.080	1.301 ± 0.065	6
trans-3-hexen-1-ol	0.241 ± 0.031	-2.000 ± 0.000	0.120 ± 0.016	5
1-pentanol	0.114 ± 0.018	-2.000 ± 0.000	0.057 ± 0.009	5
decanal	0.185 ± 0.038	-2.000 ± 0.000	0.093 ± 0.019	5
thiazole	0.087 ± 0.014	-2.000 ± 0.000	0.043 ± 0.007	5
octanoic acid	0.070 ± 0.016	-2.000 ± 0.000	-0.035 ± 0.008	5
heptanoic acid	0.054 ± 0.008	-2.000 ± 0.000	0.027 ± 0.004	5
butyric acid	0.040 ± 0.017	-2.000 ± 0.000	0.020 ± 0.008	5

L-(+)-lactic acid	0.037 ± 0.009	-2.000 ± 0.000	0.019 ± 0.004	5
methyl laurate	0.051 ± 0.014	-2.000 ± 0.000	0.026 ± 0.007	5

b)

Odorant	Activity Index	log(EC_{50})	max ΔF/F	N
eugenol	6.362 ± 0.865	-5.046 ± 0.042	1.258 ± 0.169	7
methyl benzoate	2.810 ± 0.220	-4.429 ± 0.178	0.631 ± 0.028	6
benzaldehyde	1.891 ± 0.188	-2.000 ± 0.000	0.945 ± 0.094	6
acetophenone	3.188 ± 0.405	-4.349 ± 0.060	0.732 ± 0.090	6
4-ethylphenol	3.999 ± 0.252	-4.565 ± 0.034	0.877 ± 0.059	6
2-ethylphenol	3.661 ± 0.340	-4.369 ± 0.102	0.842 ± 0.081	6
butyl acetate	1.347 ± 0.583	-3.926 ± 0.083	0.946 ± 0.136	6
ethyl hexanoate	0.631 ± 0.067	-2.000 ± 0.000	0.315 ± 0.034	6
indole	3.695 ± 0.547	-4.328 ± 0.160	0.850 ± 0.117	6
(*R*)-(+)-limonene	4.302 ± 0.519	-4.853 ± 0.099	0.882 ± 0.101	6
methyl hexanoate	3.961 ± 0.482	-3.933 ± 0.043	1.005 ± 0.119	6
4-methoxyphenylacetone	4.724 ± 0.571	-4.567 ± 0.101	1.038 ± 0.129	6
3-octanol	1.439 ± 0.175	-2.000 ± 0.000	0.632 ± 0.045	6
2-heptanone	1.650 ± 0.094	-2.000 ± 0.000	0.825 ± 0.047	6
(*R*)-1-octen-3-ol	1.588 ± 0.189	-2.000 ± 0.000	0.631 ± 0.033	6
sulcatone	1.732 ± 0.142	-2.000 ± 0.000	0.866 ± 0.071	6
1-octanol	1.937 ± 0.183	-3.870 ± 0.059	0.505 ± 0.055	6
2,4,5-trimethylthiazole	2.808 ± 0.355	-3.772 ± 0.041	0.754 ± 0.107	6
isobutyl acetate	1.656 ± 0.364	-2.000 ± 0.000	0.694 ± 0.059	6
2-acetylthiophene	2.370 ± 0.181	-4.277 ± 0.172	0.559 ± 0.050	6
isopropyl tiglate	2.953 ± 0.305	-3.934 ± 0.219	0.758 ± 0.092	5
o-cresol	1.696 ± 0.162	-2.000 ± 0.000	0.848 ± 0.081	6
hexanal	1.603 ± 0.164	-2.000 ± 0.000	0.801 ± 0.082	5
1-hexanol	0.708 ± 0.083	-2.000 ± 0.000	0.354 ± 0.041	5
prenyl acetate	1.614 ± 0.193	-2.000 ± 0.000	0.807 ± 0.096	6
alpha-pinene	4.274 ± 0.499	-4.598 ± 0.096	0.938 ± 0.122	5
ethyl butyrate	1.107 ± 0.216	-2.000 ± 0.000	0.554 ± 0.108	6
linalool	2.723 ± 0.268	-3.996 ± 0.060	0.685 ± 0.074	6
propyl acetate	0.822 ± 0.218	-2.000 ± 0.000	0.411 ± 0.109	5
(±)-geosmin	4.259 ± 0.425	-5.202 ± 0.064	0.816 ± 0.074	6
DEET	0.058 ± 0.019	-2.000 ± 0.000	0.021 ± 0.004	6
trans-3-hexen-1-ol	0.260 ± 0.026	-2.000 ± 0.000	0.130 ± 0.013	5
1-pentanol	0.183 ± 0.023	-2.000 ± 0.000	0.092 ± 0.011	5
decanal	0.207 ± 0.031	-2.000 ± 0.000	0.104 ± 0.015	5
thiazole	0.207 ± 0.064	-2.000 ± 0.000	0.104 ± 0.032	5
octanoic acid	0.071 ± 0.012	-2.000 ± 0.000	0.035 ± 0.006	5

heptanoic acid	0.262 ± 0.173	-2.000 ± 0.000	0.131 ± 0.086	5
butyric acid	0.065 ± 0.016	-2.000 ± 0.000	0.032 ± 0.008	5
L-(+)-lactic acid	0.061 ± 0.008	-2.000 ± 0.000	0.031 ± 0.004	5
methyl laurate	0.054 ± 0.014	-2.000 ± 0.000	0.027 ± 0.007	6

Supplementary Table 5. a, b. Response of MhOR5 M209V (**a**) and MhOR5 I213M (**b**) receptors to a panel of 40 odorants in the functional GCaMP assay (Fig. 4, Extended Data Fig. 9). Odorants are ordered according to how they appear in the figures (sorted left to right by the MhOR5 WT Activity Index). All values are shown with SEM. Max ΔF/F is normalized to MhOR5 with eugenol.

Ligand	CAS #	Activity Index	log(EC_{50})	max ΔF/F	N
1-hexanol	111-27-3	1.262 ± 0.175	-2.000 ± 0.000	0.631 ± 0.088	4
1-octanol	111-87-5	8.261 ± 0.414	-4.010 ± 0.039	2.057 ± 0.087	7
(*R*)-1-octen-3-ol	3391-86-4	0.500 ± 0.083	-2.000 ± 0.000	0.250 ± 0.042	4
1-pentanol	71-41-0	0.366 ± 0.069	-2.000 ± 0.000	0.183 ± 0.035	4
2-acetylthiophene	88-15-3	5.139 ± 0.383	-3.635 ± 0.122	1.421 ± 0.116	5
2-ethylphenol	90-00-6	1.087 ± 0.281	-2.000 ± 0.000	0.543 ± 0.141	3
2-heptanone	110-43-0	0.843 ± 0.191	-2.000 ± 0.000	0.421 ± 0.095	3
2-undecanone	112-12-9	0.225 ± 0.149	-2.000 ± 0.000	0.113 ± 0.074	3
2,3-butanediol	513-85-9	0.063 ± 0.022	-2.000 ± 0.000	0.032 ± 0.011	3
2,4,5-trimethylthiazole	13623-11-5	4.137 ± 0.330	-3.436 ± 0.101	1.200 ± 0.076	5
3-octanol	589-98-0	0.155 ± 0.039	-2.000 ± 0.000	0.078 ± 0.019	4
4-ethylphenol	123-07-9	0.917 ± 0.184	-2.000 ± 0.000	0.458 ± 0.092	3
4-methoxyphenylacetone	122-84-9	0.882 ± 0.121	-2.000 ± 0.000	0.441 ± 0.061	4
acetaldehyde diethyl acetal	105-57-7	0.168 ± 0.021	-2.000 ± 0.000	0.084 ± 0.010	3
acetic acid	64-19-7	0.089 ± 0.040	-2.000 ± 0.000	0.045 ± 0.020	3
acetophenone	98-86-2	5.300 ± 0.626	-3.517 ± 0.041	1.503 ± 0.167	6
alpha-pinene	7785-70-8	1.469 ± 0.296	-2.000 ± 0.000	0.735 ± 0.148	3
L-(+)-arabinose	5328-37-0	0.093 ± 0.052	-2.000 ± 0.000	0.046 ± 0.026	3
benzaldehyde	100-52-7	6.364 ± 0.859	-4.006 ± 0.068	1.598 ± 0.226	8
butyl acetate	123-86-4	0.806 ± 0.097	-2.000 ± 0.000	0.403 ± 0.049	3
butyric acid	107-92-6	0.093 ± 0.035	-2.000 ± 0.000	0.046 ± 0.018	3
caffeine	58-08-2	0.120 ± 0.067	-2.000 ± 0.000	0.060 ± 0.034	3
citric acid	77-92-9	0.075 ± 0.047	-2.000 ± 0.000	0.037 ± 0.024	3
decanal	112-31-2	0.308 ± 0.122	-2.000 ± 0.000	0.154 ± 0.061	3
DEET	134-62-3	0.067 ± 0.063	-2.000 ± 0.000	0.034 ± 0.032	3
denatonium benzoate	3734-33-6	0.492 ± 0.263	-2.000 ± 0.000	0.246 ± 0.132	4
ethyl acetate	141-78-6	0.140 ± 0.024	-2.000 ± 0.000	0.070 ± 0.012	4
ethyl butyrate	105-54-4	0.139 ± 0.033	-2.000 ± 0.000	0.069 ± 0.016	3
ethyl hexanoate	123-66-0	0.105 ± 0.057	-2.000 ± 0.000	0.052 ± 0.029	3
eugenol	97-53-0	3.988 ± 0.065	-4.082 ± 0.027	1.000 ± 0.000	21
(±)-geosmin	16423-19-1	0.033 ± 0.008	-2.000 ± 0.000	0.016 ± 0.004	4
D-(+)-glucose	50-99-7	0.085 ± 0.042	-2.000 ± 0.000	0.043 ± 0.021	4
heptanoic acid	111-14-8	0.088 ± 0.027	-2.000 ± 0.000	0.044 ± 0.013	4
hexanal	66-25-1	0.985 ± 0.095	-2.000 ± 0.000	0.492 ± 0.047	3
indole	120-72-9	4.138 ± 0.337	-3.837 ± 0.070	1.074 ± 0.073	6
isobutyl acetate	110-19-0	1.014 ± 0.236	-2.000 ± 0.000	0.507 ± 0.118	6
isopropyl tiglate	1733-25-1	0.068 ± 0.020	-2.000 ± 0.000	0.034 ± 0.010	4
L-(+)-lactic acid	79-33-4	0.114 ± 0.029	-2.000 ± 0.000	0.057 ± 0.014	4
(*R*)-(+)-limonene	5989-27-5	7.238 ± 0.318	-3.950 ± 0.023	1.832 ± 0.079	7
linalool	78-70-6	0.443 ± 0.049	-2.000 ± 0.000	0.222 ± 0.025	3
methyl benzoate	93-58-3	0.547 ± 0.045	-2.000 ± 0.000	0.274 ± 0.022	3
methyl hexanoate	106-70-7	0.335 ± 0.113	-2.000 ± 0.000	0.167 ± 0.056	3
methyl laurate	111-82-0	0.080 ± 0.023	-2.000 ± 0.000	0.040 ± 0.011	4

L-glutamic acid monosodium salt monohydrate (MSG)	6106-04-3	0.076 ± 0.056	-2.000 ± 0.000	0.038 ± 0.028	3
n-caproic acid	142-62-1	0.066 ± 0.032	-2.000 ± 0.000	0.033 ± 0.016	4
o-cresol	95-48-7	0.452 ± 0.136	-2.000 ± 0.000	0.226 ± 0.068	4
octanoic acid	124-07-2	0.101 ± 0.046	-2.000 ± 0.000	0.050 ± 0.023	4
prenyl acetate	1191-16-8	0.241 ± 0.085	-2.000 ± 0.000	0.120 ± 0.042	4
propyl acetate	109-60-4	0.210 ± 0.058	-2.000 ± 0.000	0.105 ± 0.029	4
sucrose	57-50-1	0.085 ± 0.064	-2.000 ± 0.000	0.043 ± 0.032	3
sulcatone	110-93-0	0.634 ± 0.195	-2.000 ± 0.000	0.317 ± 0.097	3
thiazole	288-47-1	0.162 ± 0.024	-2.000 ± 0.000	0.081 ± 0.012	4
trans-3-hexen-1-ol	928-97-2	0.243 ± 0.036	-2.000 ± 0.000	0.122 ± 0.018	4
VUAA1	525582-84-7	0.196 ± 0.038	-2.000 ± 0.000	0.019 ± 0.098	4

Supplementary Table 6. Response of MhOR1 to a panel of odorants, tastants and synthetic ligands in the functional GCaMP assay (Extended Data Fig. 1). All values are shown with SEM.

Odorant Receptor	# odorants	Lifetime Sparseness Value
M. hrabei OR5	54	0.346
M. hrabei OR1	54	0.764
D. melanogaster OR1a	30	0.458
D. melanogaster OR2a	124	0.607
D. melanogaster OR7a	222	0.657
D. melanogaster OR9a	144	0.546
D. melanogaster OR10a	235	0.771
D. melanogaster OR13a	167	0.606
D. melanogaster OR19a	497	0.817
D. melanogaster OR22a	225	0.585
D. melanogaster OR22b	11	0.526
D. melanogaster OR23a	115	0.757
D. melanogaster OR30a	30	0.811
D. melanogaster OR33a	30	0.280
D. melanogaster OR33b	122	0.761
D. melanogaster OR33c	12	0.472
D. melanogaster OR35a	123	0.673
D. melanogaster OR42a	72	0.702
D. melanogaster OR42b	177	0.795
D. melanogaster OR43a	115	0.793
D. melanogaster OR43b	144	0.670
D. melanogaster OR45a	31	0.480
D. melanogaster OR45b	30	0.812
D. melanogaster OR46a	12	0.983
D. melanogaster OR47a	135	0.716
D. melanogaster OR47b	178	0.872
D. melanogaster OR49a	30	0.477
D. melanogaster OR49b	164	0.880
D. melanogaster OR59a	29	0.662
D. melanogaster OR59b	173	0.810
D. melanogaster OR59c	53	0.697
D. melanogaster OR65a	116	0.754
D. melanogaster OR67a	127	0.583
D. melanogaster OR67b	121	0.522
D. melanogaster OR67c	161	0.716
D. melanogaster OR69a	107	0.149
D. melanogaster OR71a	149	0.790
D. melanogaster OR74a	30	0.619
D. melanogaster OR82a	180	0.808
D. melanogaster OR83c	125	0.667
D. melanogaster OR85a	114	0.850
D. melanogaster OR85b	161	0.620
D. melanogaster OR85c	30	0.622
D. melanogaster OR85d	51	0.809
D. melanogaster OR85e	12	0.531

D. melanogaster OR85f	114	0.473
D. melanogaster OR88a	115	0.878
D. melanogaster OR92a	174	0.853
D. melanogaster OR94a	30	0.863
D. melanogaster OR94b	31	0.602
D. melanogaster OR98a	161	0.673

Supplementary Table 7. Lifetime sparseness values for *M. hrabei* OR5 and OR1 and *D. melanogaster* ORs from published data (Extended Data Fig. 1). Those values closest to 0 correspond to broadly tuned receptors that respond similarly to many ligands in a set, while those closest to 1 are narrowly tuned to a single or to a small subset of ligands. All inhibitory responses to odorants set to 0 before calculation.

Order	a) Activity	b) ΔF/F	c) - log(EC_{50})
1	eugenol	benzaldehyde	eugenol
2	methyl benzoate	2-ethylphenol	(*R*)-(+)-limonene
3	benzaldehyde	butyl acetate	acetophenone
4	acetophenone	methyl benzoate	methyl benzoate
5	4-ethylphenol	ethyl butyrate	2-acetylthiophene
6	2-ethylphenol	4-ethylphenol	4-ethylphenol
7	butyl acetate	eugenol	ethyl hexanoate
8	ethyl hexanoate	4-methoxyphenylacetone	indole
9	indole	ethyl hexanoate	methyl hexanoate
10	(*R*)-(+)-limonene	sulcatone	2-ethylphenol
11	methyl hexanoate	indole	butyl acetate
12	4-methoxyphenylacetone	propyl acetate	3-octanol
13	3-octanol	1-hexanol	1-octanol
14	2-heptanone	alpha-pinene	benzaldehyde
15	(*R*)-1-octen-3-ol	3-octanol	2-heptanone
16	sulcatone	methyl hexanoate	(*R*)-1-octen-3-ol
17	1-octanol	isobutyl acetate	4-methoxyphenylacetone
18	2,4,5-trimethylthiazole	acetophenone	2,4,5-trimethylthiazole
19	isobutyl acetate	2-heptanone	sulcatone
20	2-acetylthiophene	2,4,5-trimethylthiazole	isobutyl acetate
21	isopropyl tiglate	(*R*)-1-octen-3-ol	isopropyl tiglate
22	o-cresol	hexanal	linalool
23	hexanal	o-cresol	o-cresol
24	1-hexanol	1-octanol	prenyl acetate
25	prenyl acetate	isopropyl tiglate	hexanal
26	alpha-pinene	prenyl acetate	1-hexanol
27	ethyl butyrate	linalool	alpha-pinene
28	linalool	(*R*)-(+)-limonene	(±)-geosmin
29	propyl acetate	2-acetylthiophene	DEET
30	(±)-geosmin	*trans*-3-hexen-1-ol	propyl acetate
31	DEET	(±)-geosmin	ethyl butyrate
32	*trans*-3-hexen-1-ol	DEET	1-pentanol
33	1-pentanol	1-pentanol	2-undecanone
34	decanal	decanal	acetaldehyde diethyl acetal
35	acetaldehyde diethyl acetal	acetaldehyde diethyl acetal	*L*-(+)-arabinose
36	thiazole	thiazole	caffeine
37	octanoic acid	octanoic acid	decanal
38	ethyl acetate	ethyl acetate	*D*-(+)-glucose
39	n-caproic acid	n-caproic acid	L-glutamic acid monosodium salt monohydrate (MSG)
40	heptanoic acid	heptanoic acid	*L*-(+)-lactic acid
41	butyric acid	butyric acid	octanoic acid
42	2-undecanone	2-undecanone	thiazole
43	*L*-(+)-lactic acid	*L*-(+)-lactic acid	2,3-butanediol
44	2,3-butanediol	2,3-butanediol	acetic acid
45	methyl laurate	methyl laurate	butyric acid
46	L-glutamic acid monosodium salt monohydrate (MSG)	L-glutamic acid monosodium salt monohydrate (MSG)	citric acid
47	acetic acid	acetic acid	methyl laurate

48	*D*-(+)-glucose	*D*-(+)-glucose	ethyl acetate
49	sucrose	sucrose	heptanoic acid
50	citric acid	citric acid	n-caproic acid
51	*L*-(+)-arabinose	*L*-(+)-arabinose	sucrose
52	caffeine	caffeine	*trans*-3-hexen-1-ol
53	denatonium benzoate	denatonium benzoate	denatonium benzoate
54	VUAA1	VUAA1	VUAA1

Supplementary Table 8. Ordering of compounds in MhOR5 tuning curves in Extended Data Fig. 2.

Ligand	1	2	3	4	5	6	7	8	9	10	11
1-hexanol	151.07	102.17	2	1	1	2	4	20.2	5.9	0.93	12.4
1-octanol	181.17	130.23	3	1	1	2	6	20.2	0.5	0.08	16.1
(*R*)-1-octen-3-ol	177.67	128.21	2.6	1	1	2	5	20.2	1.93	0.3	16.0
1-pentanol	132.42	88.15	1.6	1	1	2	3	20.2	22	2.2	10.6
2-acetylthiophene	134.58	126.18	1.2	0	2	2	1	45.3	14	3.72	13.7
2-ethylphenol	147.69	122.16	2.5	1	1	2	1	20.2	3	0.15	14.9
2-heptanone	161.14	114.19	2	0	1	1	4	17.1	4.3	3.85	13.7
2-undecanone	219.98	170.29	4.1	0	1	1	8	17.1	0.02	0.04	21.0
2,3-butanediol	117.17	90.12	-0.9	2	2	4	1	40.5	1000	0.24	9.34
2,4,5-trimethylthiazole	147.8	127.21	2.2	0	2	2	0	41.1	0.53	3.76	14.7
3-octanol	117.17	130.23	2.8	1	1	2	5	20.2	1.3	0.51	16.1
4-ethylphenol	149.23	122.16	2.6	1	1	2	1	20.2	7.4	0.04	14.9
4-methoxyphenylacetone	189.56	164.2	1.6	0	2	2	3	26.3	3.08	0.008	18.7
acetaldehyde diethyl acetal	157.28	118.18	0.8	0	2	2	4	18.5	44	27.6	13.2
acetophenone	143.45	120.15	1.6	0	1	1	1	17.1	6.13	0.4	14.4
alpha-pinene	169.51	136.23	2.8	0	0	0	0	0	0.0025	4.75	17.4
L-(+)-arabinose	141.3	150.13	-2.5	4	5	9	0	90.2	500	6.2×10^{-9}	12.5
benzaldehyde	126.34	106.12	1.5	0	1	1	1	17.1	6.95	1.27	13.1
butyl acetate	153.06	116.16	1.8	0	2	2	4	26.3	5.3	11	12.5
butyric acid	111.45	88.11	0.8	1	2	3	2	37.3	60	1.65	8.78
caffeine	187.53	194.19	-0.1	0	3	3	0	58.4	21.6	9×10^{-7}	20.0
decanal	204.92	156.26	3.8	0	1	1	8	17.1	0.029	0.1	19.3
DEET	220.87	191.27	2	0	1	1	3	20.3	0.91	0.002	23.3
ethyl acetate	117.6	88.11	0.7	0	2	2	2	26.3	80	93.2	8.86
ethyl butyrate	155.12	116.16	1.3	0	2	2	4	26.3	4.9	14	12.5
ethyl hexanoate	188.21	144.21	2.4	0	2	2	6	26.3	0.63	1.78	16.2
eugenol	189.2	164.2	2	1	2	3	3	29.5	2.46	0.02	19.3
(±)-geosmin	196.36	182.3	3.3	1	1	2	0	20.2	0.157	0.003	21.8
D-(+) glucose	165.56	180.16	-2.6	5	6	11	1	110	960	5.9×10^{-11}	14.9
heptanoic acid	164.52	130.18	2.5	1	2	3	5	37.3	2.82	0.01	14.3
hexanal	143.01	100.16	1.8	0	1	1	4	17.1	5.64	11.3	11.9
indole	134.94	117.15	2.1	1	0	1	0	15.8	3.56	0.01	15.3
isobutyl acetate	189.2	116.16	1.8	0	2	2	3	26.3	6.3	17.8	12.5
isopropyl tiglate	180.68	142.2	2.1	0	2	2	3	26.3	0.5	3.51	16.2
L-(+)-lactic acid	101.64	90.08	-0.7	2	3	5	1	57.5	1000	0.08	7.53
(*R*)-(+)-limonene	179.13	136.25	3.4	0	0	0	1	0	0.007	1.64	18.0
linalool	200.28	154.25	2.7	1	1	2	4	20.2	1.59	0.16	19.6
methyl benzoate	154.73	136.15	2.1	0	2	2	2	26.3	2.1	0.38	15.1
methyl hexanoate	170.24	130.18	2.5	0	2	2	5	26.3	1.33	3.72	14.4
methyl laurate	278.56	214.34	5.8	0	2	2	11	26.3	0.013	4.11×10^{-3}	25.4
n-caproic acid	147.04	116.16	1.9	1	2	3	4	37.3	10.3	0.04	12.5
o-cresol	130.17	108.14	2	1	1	2	0	20.2	25.9	0.3	13.1
octanoic acid	180.9	144.21	3	1	2	3	6	37.3	0.789	3.71×10^{-3}	16.1
prenyl acetate	164.81	128.17	1.8	0	2	2	3	26.3	3.2	5.4	14.3
propyl acetate	138.16	102.13	1.2	0	2	2	3	26.3	18.9	35.9	10.7
sulcatone	171.92	126.2	1.9	0	1	1	3	17.1	3.2	0.008	15.5
thiazole	90.71	85.13	0.4	0	2	2	0	41.1	53	17.15	9.01
trans-3-hexen-1-ol	144.57	100.16	1.3	1	1	2	3	20.2	16	1.36	12.5

Supplementary Table 9. Compilation of molecular descriptors of the ligands used in multiple regression analysis (Extended Data Table 2). 1: Area, 2: Molecular weight (g/mol), 3: estimated octanol/water partition coefficient (XlogP3-AA), 4: Hydrogen bond count, donor, 5: Hydrogen bond count, acceptor, 6: Hydrogen bond count, total, 7:

Rotatable bond count, 8: Polar surface area (Å^2), 9: Water solubility (g/L), 10: Vapor pressure (mm Hg), 11: Polarizability (Å^3). Sources: PubChem, Sigma-Aldrich, ChemSpider, EPA, and The Good Scents Company.

a. eugenol

Mutation	Activity Index	log(EC_{50})	max $\Delta F/F$	N
wild-type MhOR1	4.103 ± 0.033	-4.103 ± 0.033	1.000 ± 0.000	9
L102A	4.657 ± 0.455	-3.758 ± 0.120	1.234 ± 0.082	3
T103A	0.127 ± 0.034	-2.000 ± 0.000	0.064 ± 0.017	3
Y106A	0.121 ± 0.031	-2.000 ± 0.000	0.060 ± 0.015	3
I107A	0.135 ± 0.024	-2.000 ± 0.000	0.067 ± 0.012	3
S166A	0.220 ± 0.030	-2.000 ± 0.000	0.110 ± 0.015	3
G169A	-0.123 ± 0.011	-2.000 ± 0.000	-0.062 ± 0.006	3
W173A	0.115 ± 0.006	-2.000 ± 0.000	0.057 ± 0.003	3
L227A	0.098 ± 0.020	-2.000 ± 0.000	0.049 ± 0.010	3
M231A	0.121 ± 0.014	-2.000 ± 0.000	0.060 ± 0.007	3
L398A	1.570 ± 0.374	-4.697 ± 0.014	0.334 ± 0.079	3
Y399A	0.346 ± 0.061	-2.000 ± 0.000	0.173 ± 0.031	3
C402A	4.002 ± 0.866	-4.163 ± 0.079	0.967 ± 0.217	3

b. 1-octanol

Mutation	Activity Index	log(EC_{50})	max $\Delta F/F$	N
wild-type MhOR1	7.858 ± 0.361	-4.240 ± 0.061	1.856 ± 0.100	4
L102A	6.628 ± 0.587	-4.249 ± 0.017	1.559 ± 0.133	3
T103A	0.371 ± 0.129	-2.000 ± 0.000	0.185 ± 0.065	3
Y106A	0.127 ± 0.024	-2.000 ± 0.000	0.063 ± 0.012	3
I107A	0.188 ± 0.103	-2.000 ± 0.000	0.094 ± 0.051	3
S166A	0.117 ± 0.070	-2.000 ± 0.000	0.059 ± 0.035	3
G169A	2.019 ± 0.049	-5.403 ± 0.025	0.374 ± 0.010	3
W173A	0.123 ± 0.085	-2.000 ± 0.000	0.061 ± 0.042	3
L227A	4.488 ± 0.916	-3.347 ± 0.030	1.344 ± 0.283	3
M231A	0.133 ± 0.080	-2.000 ± 0.000	0.066 ± 0.040	3
L398A	2.669 ± 0.817	-4.684 ± 0.343	0.581 ± 0.177	3
Y399A	0.245 ± 0.025	-2.000 ± 0.000	0.123 ± 0.012	3
C402A	6.906 ± 1.381	-4.841 ± 0.145	1.430 ± 0.284	3

Supplementary Table 10. Response of wild-type and putative binding-pocket mutant MhOR1 receptors in the functional GCaMP assay (Extended Data Fig. 11) to eugenol (**a**) and 1-octanol (**b**). All values are shown with SEM.

a. wild-type MhOR1

Odorant	Activity Index	log(EC_{50})	N
benzaldehyde	7.938 ± 1.299	-3.797 ± 0.181	4
1-octanol	7.779 ± 0.389	-4.237 ± 0.022	4
2-acetylthiophene	6.701 ± 0.485	-3.808 ± 0.019	4
(*R*)-(+)-limonene	6.586 ± 0.203	-3.980 ± 0.051	4
acetophenone	5.742 ± 0.671	-3.595 ± 0.025	3
2,4,5-trimethylthiazole	4.534 ± 0.364	-3.408 ± 0.122	3
eugenol	4.058 ± 0.021	-4.087 ± 0.021	15
indole	2.450 ± 0.086	-2.000 ± 0.000	4
2-ethylphenol	0.970 ± 0.169	-2.000 ± 0.000	4
4-ethylphenol	0.910 ± 0.125	-2.000 ± 0.000	4
4-methoxyphenylacetone	0.682 ± 0.056	-2.000 ± 0.000	4
sulcatone	0.509 ± 0.070	-2.000 ± 0.000	3
methyl benzoate	0.498 ± 0.046	-2.000 ± 0.000	4
methyl hexanoate	0.247 ± 0.039	-2.000 ± 0.000	3
prenyl acetate	0.133 ± 0.017	-2.000 ± 0.000	3
(±)-geosmin	0.039 ± 0.013	-2.000 ± 0.000	3
DEET	0.020 ± 0.010	-2.000 ± 0.000	4

b. MhOR1 M231I

Odorant	Activity Index	log(EC_{50})	N
benzaldehyde	9.436 ± 1.224	-4.313 ± 0.100	4
1-octanol	8.454 ± 0.527	-4.446 ± 0.037	3
2-acetylthiophene	8.192 ± 0.526	-4.324 ± 0.050	5
(*R*)-(+)-limonene	9.004 ± 0.563	-4.174 ± 0.093	4
acetophenone	9.350 ± 0.916	-4.337 ± 0.051	5
2,4,5-trimethylthiazole	7.841 ± 1.020	-3.663 ± 0.071	3
eugenol	6.105 ± 0.220	-4.190 ± 0.022	7
indole	7.699 ± 0.418	-3.735 ± 0.073	4
2-ethylphenol	4.967 ± 0.223	-3.489 ± 0.016	4
4-ethylphenol	3.471 ± 0.346	-3.452 ± 0.110	3
4-methoxyphenylacetone	5.497 ± 0.273	-3.658 ± 0.079	4
sulcatone	1.483 ± 0.099	-2.000 ± 0.000	3
methyl benzoate	7.111 ± 0.626	-2.000 ± 0.000	4
methyl hexanoate	1.895 ± 0.440	-2.000 ± 0.000	3
prenyl acetate	1.493 ± 0.293	-2.000 ± 0.000	3
(±)-geosmin	0.019 ± 0.021	-2.000 ± 0.000	3
DEET	0.071 ± 0.012	-2.000 ± 0.000	4

Supplementary Table 11. Wild-type MhOR1 (**a**) and MhOR1 M231I (**b**) tuning curves in response to a panel of 17 ligands in the functional GCaMP assay (Extended Data Fig. 11). The curves are sorted left to right according to the highest to lowest scoring wild-type MhOR1 responses and match the ordering featured in Extended Data Fig. 12. All values are shown with SEM.
