# Supplementary material for: The structural basis of odorant recognition in insect olfactory receptors
Source: Nature. 2021 Aug 4;597(7874):126–31. doi: 10.1038/s41586-021-03794-8 (PMC8410599; doi:10.1038/s41586-021-03794-8)
Supplement: Supplementary file 2 — Reporting Summary [file 41586_2021_3794_MOESM2_ESM.pdf]

nature research

Corresponding author(s): Vanessa Ruta

Last updated by author(s): 06/20/2021

Reporting Summary

Nature Research wishes to improve the reproducibility of the work that we publish. This form provides structure for consistency and transparency in reporting. For further information on Nature Research policies, see our Editorial Policies and the Editorial Policy Checklist.

Statistics

For all statistical analyses, confirm that the following items are present in the figure legend, table legend, main text, or Methods section.

n/a	Confirmed	
☐	☒	The exact sample size (n) for each experimental group/condition, given as a discrete number and unit of measurement
☐	☒	A statement on whether measurements were taken from distinct samples or whether the same sample was measured repeatedly
☐	☒	The statistical test(s) used AND whether they are one- or two-sided<br>*Only common tests should be described solely by name; describe more complex techniques in the Methods section.*
☒	☐	A description of all covariates tested
☐	☒	A description of any assumptions or corrections, such as tests of normality and adjustment for multiple comparisons
☐	☒	A full description of the statistical parameters including central tendency (e.g. means) or other basic estimates (e.g. regression coefficient) AND variation (e.g. standard deviation) or associated estimates of uncertainty (e.g. confidence intervals)
☐	☒	For null hypothesis testing, the test statistic (e.g. F, t, r) with confidence intervals, effect sizes, degrees of freedom and P value noted<br>*Give P values as exact values whenever suitable.*
☒	☐	For Bayesian analysis, information on the choice of priors and Markov chain Monte Carlo settings
☒	☐	For hierarchical and complex designs, identification of the appropriate level for tests and full reporting of outcomes
☐	☒	Estimates of effect sizes (e.g. Cohen's d, Pearson's r), indicating how they were calculated

Our web collection on statistics for biologists contains articles on many of the points above.

Software and code

Policy information about availability of computer code

Data collection	For cryo-EM collection: SerialEM; for electrophysiology data: Clampex 10.6.
Data analysis	Relion-3.0, cryoSPARCv2, PyMOL, Chimera, ChimeraX, Schrodinger Maestro, GraphPad Prism, Coot, MotionCor2, CTFFIND4, PHENIX, HOLE, JalView

For manuscripts utilizing custom algorithms or software that are central to the research but not yet described in published literature, software must be made available to editors and reviewers. We strongly encourage code deposition in a community repository (e.g. GitHub). See the Nature Research guidelines for submitting code & software for further information.

Data

Policy information about availability of data

All manuscripts must include a data availability statement. This statement should provide the following information, where applicable:

- Accession codes, unique identifiers, or web links for publicly available datasets
- A list of figures that have associated raw data
- A description of any restrictions on data availability

The 3D cryo-EM density map of unbound MhOR5, eugenol-bound MhOR5, and DEET-bound MhOR5 have been deposited in the Electron Microscopy Data Bank under accession numbers EMD-23372, EMD-23374, and EMD-23375, respectively. The coordinates of the atomic models of unbound MhOR5, eugenol-bound MhOR5, and DEET-bound MhOR5 have been deposited in the Protein Data Bank under accession numbers 7LIC, 7LID and 7LIG, respectively. Raw data associated with functional analyses in Fig. 1c,2e,2f,3d,4d,4e and Extended Data Fig. 1d,1f,2,10a-c,11c is available upon request and summarized in Tables 2,4,6-9.

Field-specific reporting

Please select the one below that is the best fit for your research. If you are not sure, read the appropriate sections before making your selection.

☒ Life sciences ☐ Behavioural & social sciences ☐ Ecological, evolutionary & environmental sciences

For a reference copy of the document with all sections, see nature.com/documents/nr-reporting-summary-flat.pdf

Life sciences study design

All studies must disclose on these points even when the disclosure is negative.

Sample size	No calculations were performed to determine sample sizes; however, the addition of more data did not alter conclusions from this study.
Data exclusions	For processing cryo-EM data, some particles were excluded following standard procedures and described extensively in the methods section.
Replication	Functional experiments were repeated on different days, using independently transfected cells and independently prepared odorant solutions. Cryo-EM datasets were collected on 4 different days: the apo structure comes from 2 datasets collected on independent days, and the eugenol and DEET structures come from one single dataset each.
Randomization	This study did not allocate experimental groups; thus, no randomization was necessary.
Blinding	No blinding was used; all functional data were analyzed using the same methods and all data were included in the results.

Reporting for specific materials, systems and methods

We require information from authors about some types of materials, experimental systems and methods used in many studies. Here, indicate whether each material, system or method listed is relevant to your study. If you are not sure if a list item applies to your research, read the appropriate section before selecting a response.

Materials & experimental systems

n/a	Involved in the study	
☒	☐	Antibodies
☐	☒	Eukaryotic cell lines
☒	☐	Palaeontology and archaeology
☒	☐	Animals and other organisms
☒	☐	Human research participants
☒	☐	Clinical data
☒	☐	Dual use research of concern

Methods

n/a	Involved in the study	
☒	☐	ChIP-seq
☒	☐	Flow cytometry
☒	☐	MRI-based neuroimaging

Eukaryotic cell lines

Policy information about cell lines

Cell line source(s)	Sf9 (ATCC CRL-1711). HEK293S GnTi- (ATCC CRL-3022).
Authentication	None were authenticated.
Mycoplasma contamination	Cells were tested for mycoplasma and no contamination was detected.
Commonly misidentified lines (See ICLAC register)	N/A.
